# Sequencing and analysis of the mitochondrial genome of *Pennahia anea* (Bloch, 1793) (Perciformes: Sciaenidae)

**DOI:** 10.1080/23802359.2021.1910084

**Published:** 2021-04-15

**Authors:** Thinh Dinh Do, Chien Pham Van, Minh Dong Dao, Thanh Vu Quyet, Van Quan Nguyen, Chang-Bae Kim

**Affiliations:** a Institute of Marine Environment and Resources, Vietnam Academy of Science and Technology, Hanoi, Vietnam; b Department of Biotechnology, Sangmyung University, Seoul, Korea; c Graduate School of Science and Technology, Hanoi, Vietnam; d Bach Long Vy’s people committee, Haiphong, Vietnam; e Vietnam Russia Tropical Center, Hanoi, Vietnam

**Keywords:** Mitogenome, croaker, *Pennahia anea*, phylogeny

## Abstract

Donkey croaker, *Pennahia anea* (Bloch, 1793) is a commercially important croaker in the Indo-Pacific region. In this study, we sequenced and analyzed the mitogenome of *P. anea*. The nearly complete mitochondrial genome of *P. anea* is 15,694 bp in size. It contained 13 protein-coding genes (PCGs), 2 rRNA genes, and 22 tRNA genes. The sequence had the A-T content of 55.4% and GC content of 44.6%. All 13 PCGs used ATG codon for initiation, while TAA codon was the most common for termination. Phylogenetic analysis demonstrated that *P. anea* is located within the genus *Pennahia*. This study provides additional data for the understanding of the phylogeny of the family Sciaenidae.

Sciaenidae is a family of the order Perciformes that contains around 300 species worldwide (Quan et al. 2011; Froese and Pauly 2019). Some species of this family are used as food fish and are important targets for commercial fishery. The ability to produce a croaking sound is known as a distinguishing characteristic of sciaenids (Ramcharitar et al. 2006). *Pennahia anea* is one of five species of the genus *Pennahia*, a member of the family Sciaenidae. This species is distributed in the Indo-Pacific region, including Vietnamese waters (Froese and Pauly 2019). *P. anea* is a commercially important species in the distributed region. Of five species of the genus *Pennahia*, three have been sequenced mitogenomes, namely *Pennahia argentata*, *Pennahia macrocephalus*, and *Pennahia pawak*. Sequencing the mitogenome of the remaining species is necessary to understand phylogenetic relationships within the genus.

Specimens of *P. anea* were collected from Phu Long - Cat Hai, Vietnam (20°45'15.06"N - 106°54'53.62"E) during November 2019 to August 2020. A specimen used for mitogenome sequencing was deposited at the Institute of Marine Environment and Resources, Vietnam (contact person: Chien Pham Van, email: chienimer@gmail.com) under the voucher number IMER-TA-007. Following DNA extraction, 150 bp paired-end libraries were prepared and analyzed using the MGISEQ-2000 system (MGI, Shenzen, China). Subsequently, raw data were assembled using Mitobim program and annotation was performed using MITOS program (Bernt et al. 2013; Hahn et al. 2013). A phylogenetic tree of the family Sciaenidae was constructed based on 13 protein-coding genes (PCGs). To build the tree, Bayesian inference method in MrBayes version 3.2.7a was applied with two independent runs, 10 million generations, and sampling every 1000 generations (Ronquist et al. 2012).

Through the sequencing, the nearly complete mitogenome of *P. anea* was obtained. The nearly complete mitogenome of *P. anea* (GeneBank accession number: MW408700) was 15,694 bp long and contained the typical set of 37 genes, including 13 PCGs, and 2rRNA genes, and 22 tRNA genes. The nucleotide composition of the sequence was 27.3% A, 28.2% C, 16.4% G and 28.1% T. Base composition is similar to that observed in other Perciformes species (Ceruso, Mascolo, Lowe, et al. 2018, Ceruso, Mascolo, Palma, et al. 2018; Mascolo et al. 2018a, 2018b, 2019; Ceruso et al. 2020). The sizes of genes in the mitogenome of *P. anea* were comparable to that of other *Pennahia* species (Li et al. 2014; Guo et al. 2016; Lin et al. 2017). The sizes of PCGs ranged from 168 bp (*atp8*) to 1,815 bp (*nd5*) and the sizes of tRNA genes ranged from 68 bp (tRNA-Ser) to 75 bp (tRNA-Lys and tRNA-Leu). Two rRNA genes, 12S rRNA and 16S rRNA were 958 and 1,709 bp in size. All PCGs used ATG codon for initiation. Meanwhile, five of 13 PCGs used TAA codon for termination (*atp6*, *atp8*, *nd1*, *nd4l*, and *nd6*). AGA termination codon was observed in *cox1* and *nd4*, and TAG termination codon was observed in *nd5* while AGG termination codon was observed in *cytb* and *nd3*. The incomplete termination codon was found in *cox2*, *cox3*, and *nd2* (Miya et al 2001; Ceruso et al. 2019).

Phylogenetic analysis based on nucleotide sequences of 13 PCGs showed the position of *P. anea* in the family Sciaenidae (Figure 1). The phylogenetic tree revealed that *P. anea* was clustered with other members of the genus *Pennahia*, which was sister to *Protonibea diacanthus*. Our finding is in agreement with previous studies that showed the relationships of the genus *Pennahia* with other species of the family Sciaenidae (Lin et al. 2017; Wen et al. 2020). The mitogenome reported from this study might be helpful for future studies that deal with the genetic diversity of *P. anea*.

**Figure 1.**
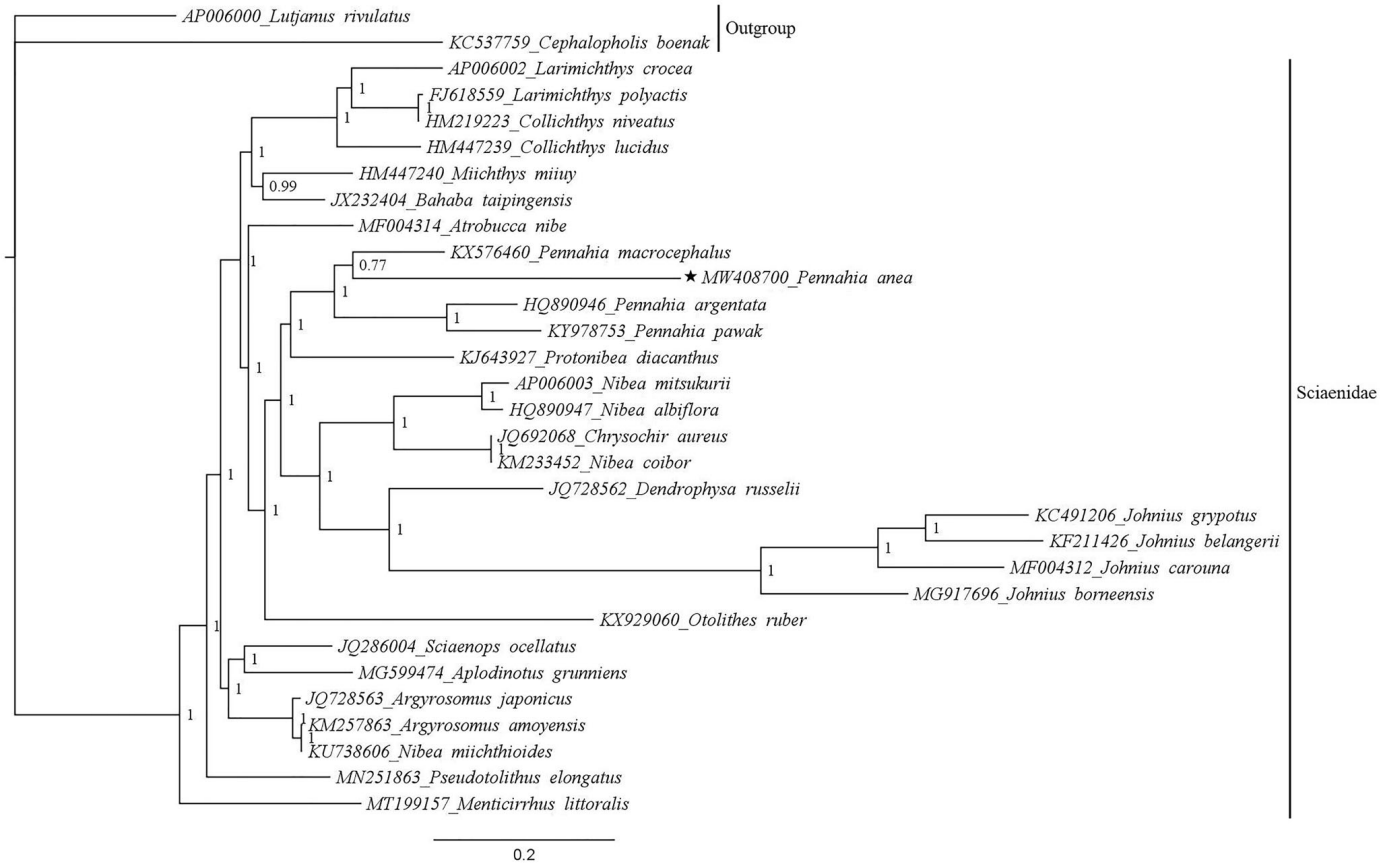
The phylogenetic position of *Pennahia anea* in the family Sciaenidae based on 13 protein-coding genes. The mitogenome of *P. anea* was marked with a star. *Lutjanus rivulatus* and *Cephalopholis boenak* represent outgroups. Numbers at nodes indicate posterior probability values.

## Data Availability

The genome sequence data that support the findings of this study is openly available in GenBank of NCBI at (https://www.ncbi.nlm.nih.gov/) under the accession no. MW408700. The associated BioProject, SRA, and Bio-Sample numbers are PRJNA694527, SRR13555243, and SAMN17376889, respectively.
